# Chemotactic Signaling by Single-Chain Chemoreceptors

**DOI:** 10.1371/journal.pone.0145267

**Published:** 2015-12-28

**Authors:** Patricia Mowery, Peter Ames, Rebecca H. Reiser, John S. Parkinson

**Affiliations:** 1 Department of Biology, University of Utah, Salt Lake City, Utah, United States of America; 2 Department of Biology, Hobart and William Smith Colleges, Geneva, New York, United States of America; Centre National de la Recherche Scientifique, Aix-Marseille Université, FRANCE

## Abstract

Bacterial chemoreceptors of the methyl-accepting chemotaxis protein (MCP) family operate in commingled clusters that enable cells to detect and track environmental chemical gradients with high sensitivity and precision. MCP homodimers of different detection specificities form mixed trimers of dimers that facilitate inter-receptor communication in core signaling complexes, which in turn assemble into a large signaling network. The two subunits of each homodimeric receptor molecule occupy different locations in the core complexes. One subunit participates in trimer-stabilizing interactions at the trimer axis, the other lies on the periphery of the trimer, where it can interact with two cytoplasmic proteins: CheA, a signaling autokinase, and CheW, which couples CheA activity to receptor control. As a possible tool for independently manipulating receptor subunits in these two structural environments, we constructed and characterized fused genes for the *E*. *coli* serine chemoreceptor Tsr that encoded single-chain receptor molecules in which the C-terminus of the first Tsr subunit was covalently connected to the N-terminus of the second with a polypeptide linker. We showed with soft agar assays and with a FRET-based *in vivo* CheA kinase assay that single-chain Tsr~Tsr molecules could promote serine sensing and chemotaxis responses. The length of the connection between the joined subunits was critical. Linkers nine residues or shorter locked the receptor in a kinase-on state, most likely by distorting the native structure of the receptor HAMP domain. Linkers 22 or more residues in length permitted near-normal Tsr function. Few single-chain molecules were found as monomer-sized proteolytic fragments in cells, indicating that covalently joined receptor subunits were responsible for mediating the signaling responses we observed. However, cysteine-directed crosslinking, spoiling by dominant-negative Tsr subunits, and rearrangement of ligand-binding site lesions revealed subunit swapping interactions that will need to be taken into account in experimental applications of single-chain chemoreceptors.

## Introduction

Motile *Escherichia coli* detect and follow gradients of attractant and repellent chemicals with high sensitivity over a broad concentration range, a behavior termed chemotaxis [1–3]. Stimulus detection and amplification occur in ternary core signaling complexes of transmembrane chemoreceptors (methyl-accepting chemotaxis proteins; MCPs), a cytoplasmic histidine kinase, CheA, and the CheW protein, which couples CheA to receptor control. Core signaling complexes form large clusters that amplify input stimuli through highly cooperative signaling behavior. The functional architecture of these receptor arrays is not yet fully understood at the molecular level.

Attractant stimuli down-regulate the autophosphorylation activity of CheA, slowing the flux of CheA-generated phosphoryl groups to the CheY response regulator [1–3]. Phospho-CheY interacts with the flagellar motors, inducing episodes of clockwise rotation that cause random directional changes or tumbles as the cell swims. When phospho-CheY levels drop, the flagellar motors rotate in the counter-clockwise default direction, producing forward swimming movements. A dedicated phosphatase, CheZ, ensures that phospho-CheY messengers are short-lived, allowing the cell to respond rapidly to changing chemical conditions as it swims about. Two MCP-specific enzymes, CheR, a methyltransferase, and CheB, a methylesterase, reversibly modify receptor molecules to tune their ligand sensitivity to ambient conditions. This sensory adaptation system enables receptor signaling arrays to operate over a 5–6 log range of ligand concentrations.

**Fig 1:**
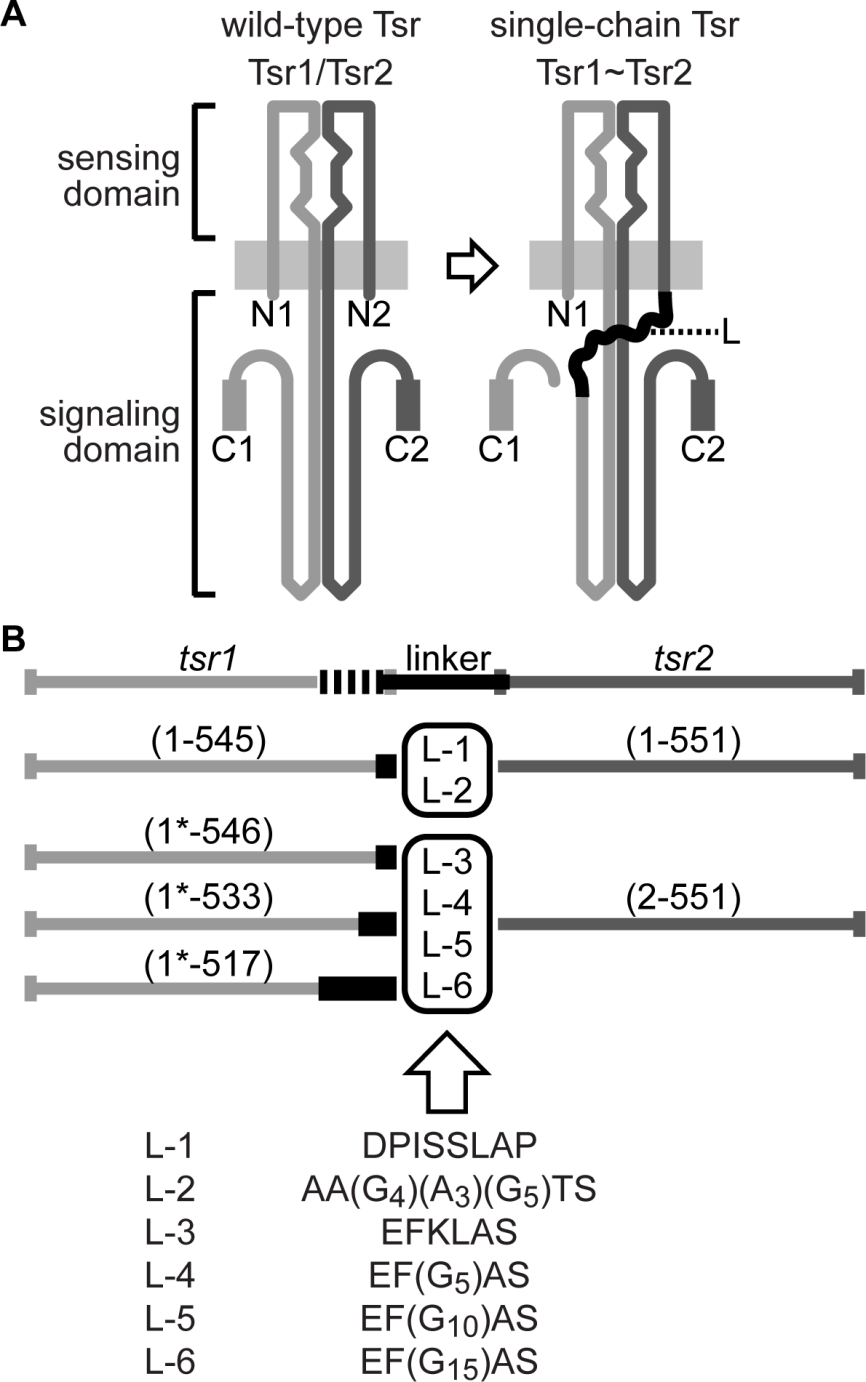
Construction scheme for single-chain Tsr molecules. (A) Schematic of a wild-type Tsr and a single-chain Tsr1~Tsr2 molecule. The gray rectangles represent the cytoplasmic membrane. The sensing domain is periplasmic; the signaling domain is cytoplasmic. The two subunits of the native homodimer are shaded light gray (Tsr1) and dark gray (Tsr2). “N” and “C” marker their N- and C-termini. “L” is a linker polypeptide that joins Tsr1 and Tsr2 in single-chain constructs. The thickened C-terminal segments represent the NWETF pentapeptide to which CheR and CheB bind. Single-chain constructs lack one of these pentapeptides. The clefts at the dimer interface of the periplasmic domain represent binding determinants of the two symmetric ligand-binding sites. (B) Summary of the various single-chain constructs made in this work. Coding regions for Tsr1 and Tsr2 are designated *tsr1* and *tsr2*, respectively, with their codon numbers in parentheses. 1* denotes one extra codon created by the cloning process. Thickened, black segments in tsr1 represent deletions of C-terminal coding sequences.

Native MCPs are homodimeric molecules that typically have a periplasmic ligand-binding domain and a cytoplasmic signaling domain (Fig 1A) [4]. *E*. *coli* has four such MCPs, the most abundant of which is Tsr, the serine receptor. The highly conserved signaling domains of MCPs organize receptor molecules into trimers of dimers [5], which can contain receptors of different types [6, 7]. Receptor core complexes, the minimal units of receptor signaling, contain two trimers of dimers, one CheA dimer, and two CheW molecules [8]. The trimer contacts between receptor molecules lie near their cytoplasmic hairpin tips, a region that also contains the binding determinants for CheA and CheW [9–15]. In trimers of dimers, one subunit of each receptor dimer resides at the dimer-dimer interface at the trimer axis, whereas the other subunits lie at the periphery of the trimer. The nearly invariant natures and dual structural environments of the trimer contact residues suggest that they play several distinct functional roles. For example, trimer contact residues in the outside orientation probably promote binding interactions with CheA and/or CheW.

Conventional mutational approaches cannot distinguish the disparate functional environments in the receptor trimer of dimers. As a possible tool for exploring the functional roles of MCP residues in the two structural environments at the trimer tip, we constructed receptor genes that encode receptor dimers that have covalently connected subunits. We reasoned that such single-chain receptors could enable us to manipulate subunit composition and orientation within a trimer of dimers. For example, we would be able to produce receptors with a structural alteration in only one subunit of the single-chain molecule or with different lesions in each subunit. In addition, these constructs mirror naturally occurring soluble single-chain MCP-like proteins found in numerous genomes [16, 17], and so they may provide insight into organization and function of these unexplored proteins. Here we report the construction and signaling properties of single-chain molecules of the *E*. *coli* serine chemoreceptor, Tsr.

## Materials and Methods

### Bacterial strains and plasmids

All strains used in this work were derivatives of *E*. *coli* K12 strain RP437 [18]. Their designations and relevant genotypes were: UU1609 [*Δ(flhD-flhA)4 Δtsr-7028 Δtrg-100 recA1*] (this work); UU2567 [*Δ(tar-cheZ)4211 Δtsr-5547 Δaer-1 Δtrg-4543*] [19]; and UU1448 [*Δtsr-7028 Δ(tar-tap)5201 Δtrg-100 recA56*] (obtained as KO607 [20] from R. Bourret, University of North Carolina Medical School).

Plasmids used in this study are listed in S1 Table.

### Construction of single-chain receptor genes

All single-chain receptor genes were constructed and manipulated on plasmids with regulatable expression control (see S1 Table). Such plasmids were generally created by PCR (polymerase chain reaction) amplification of the *tsr* gene, using primers that introduced restriction enzyme recognition sites at each end of the coding region. Most single-chain constructs were made by two sequential PCR amplification and cloning steps, using unique restriction sites at the ends of each *tsr* gene. The connecting linker oligonucleotides were inserted either during or after the *tsr* cloning steps. Plasmid pRR22 was built by creating a pRR48 [7] single-chain derivative and then subcloning a '*tsr*-L-1-*tsr*' NdeI fragment from that plasmid into the NdeI site of plasmid pJC3 [21], which lies in the middle of the *tsr* coding region. Single-chain constructs bearing mutation(s) in one or both *tsr* copies were made in a similar manner, using mutant *tsr* genes as templates for the PCR amplification(s). All single-chain constructs were verified by DNA sequence analysis.

### Soft agar chemotaxis assays

Chemotactic ability of single-chain receptor strains was assessed by colony size on tryptone semisolid agar plates [22] containing appropriate antibiotics (50 μg ml^-1^ ampicillin and/or 12.5 μg ml^-1^ chloramphenicol) and variable amounts of isopropyl-β-D-thiogalactopyranoside (IPTG) and/or sodium salicylate to induce Tsr expression. Plates were incubated at 32.5°C for 6–8.5 hours.

### Isolation of improved host strains for single-chain Tsr function

Strain UU1448 carrying plasmid pRR22 was grown to early stationary phase in tryptone broth containing 100 μg ml^-1^ ampicillin, and ~0.1 ml samples of the culture were streaked in a line across the surface of tryptone soft agar plates containing 50 μg ml^-1^ ampicillin and different concentrations of IPTG. The plates were incubated overnight at 32.5°C, and faster-spreading flares, presumably created by spontaneous mutation events, were picked and single-colony purified. The cells were cured of their plasmids by growing them for 30–40 generations in tryptone broth with no ampicillin selection and then scoring individual clones for ampicillin sensitivity. Cured derivatives were retransformed with pRR22 to assess single-chain Tsr function in the mutant background. Two independent variant strains, UU1449 and UU1450, were kept for further use.

### Protein electrophoretic analysis

Cell lysates of strains carrying Tsr plasmids were prepared and analyzed by denaturing electrophoresis in sodium dodecyl sulfate-containing polyacrylamide gels (SDS-PAGE), essentially as described [6]. Tsr proteins were visualized by immunoblotting with a polyclonal rabbit antiserum directed against the highly conserved portion of the Tsr signaling domain [23] and either ^35^S-Protein A [21] or a fluorescent secondary antibody (Cy5-goat anti-rabbit, Amersham Biosciences, Pittsburgh, PA). Western blots were imaged and quantified with a Molecular Dynamics Typhoon instrument.

### Disulfide crosslinking

Strain UU1609 carrying appropriate plasmids was grown at 30°C in tryptone broth containing 50 μg ml^-1^ ampicillin. At OD_600_ = 0.1, IPTG was added (10 μM for Tsr~Tsr; 15 μM for Tsr monomers). At OD_600_ = 0.5, the cells were centrifuged, washed, and resuspended in KPi (50 mM potassium phosphate, pH 7.0) at OD_600_ = 2. Cells were incubated at 37°C with or without 2 mM Cu(II)-(o-phenanthroline)_3_ (Cu-Phenanthroline) for 20 minutes, after which 10 mM *N*-ethylmaleimide (NEM) and 10 mM ethylenediaminetetraacetic acid (EDTA) were added to block free thiol groups and chelate copper ions, respectively. Cells were boiled in SDS sample buffer [24] containing 10 mM NEM and 10 mM EDTA and lysate proteins were analyzed by SDS-PAGE and visualized by immunoblotting as described above.

For crosslinking timecourses, samples were induced as described above, then treated with or without 500 μg ml^-1^ chloramphenicol and incubated at 30°C with shaking for 90 minutes. Samples were washed and resuspended in 50 mM KPi at OD_600_ = 2 with or without 500 μg ml^-1^ chloramphenicol, and incubated with 2 mM Cu-phenanthroline at 37°C. Samples were analyzed as described above.

### *In vivo* FRET (Förster Resonance Energy Transfer) CheA kinase assay

The experimental system, cell sample chamber, stimulus protocol, and data analysis followed the hardware, software, and methods described by Sourjik et al. [25], with minor modifications [26]. Cells containing a FRET reporter plasmid (pRZ30 [19, 26] or pVS88 [27]) and a compatible *tsr* expression plasmid (pRR53 [7] or pRR52 derivative) were grown at 30°C to mid-exponential phase in tryptone broth, washed, attached to a round coverslip with polylysine, and mounted in a flow cell [28]. The flow cell and all motility buffer test solutions [KEP containing 10 mM Na lactate, 100 μM methionine, and various concentrations of serine] were maintained at 30°C throughout each experiment. Cells were illuminated at the CFP (cyan fluorescent protein) excitation wavelength and light emission detected at the CFP (FRET donor) and YFP (FRET acceptor) wavelengths with photomultipliers. The ratio of YFP (yellow fluorescent protein) to CFP photon counts reflects CheA kinase activity and changes in response to serine stimuli [25, 27]. Fractional changes in kinase activity versus applied serine concentrations were fitted to a multi-site Hill equation, yielding two parameter values: *K*_*1/2*_, the attractant concentration that inhibits 50% of the kinase activity; and the Hill coefficient, reflecting the extent of cooperativity of the response [25, 27]. The kinase activity produced by receptors that failed to respond to serine stimuli was estimated by the drop in the FRET signal upon treatment with 3 mM potassium cyanide [26].

### Receptor clustering

Cellular receptor clusters were visualized by fluorescence light microscopy, using YFP-CheW expressed from plasmid pPA801 as the cluster reporter [29]. Tsr single-chain derivatives of compatible plasmid pPM2 were introduced into strain UU1450 carrying pPA801, and transformant cells were grown at 30°C in tryptone broth with 12.5 μg ml^-1^ chloramphenicol, 50 μg ml^-1^ ampicillin, 50 μM IPTG, and 0.004% arabinose. Cells were examined at mid-exponential phase by fluorescence microscopy as previously described [21, 30]. At least 100 cell images from each of two independent experiments were scored for the presence or absence of polar clusters.

## Results

### Construction of single-chain receptors

We created single-chain receptors by joining the C-terminus of one Tsr subunit (Tsr1) to the N-terminus of a second (Tsr2) with a polypeptide linker (Fig 1A). (To simplify the ensuing discussion, we will refer to Tsr1 and Tsr2 as “subunits,” despite their covalent connection in single-chain molecules.) The high-abundance receptors, Tsr (serine-sensing) and Tar (aspartate and maltose-sensing), carry a C-terminal pentapeptide (NWETF) that serves as a binding site for CheR [31] and, less strongly, for CheB [32]. To avoid steric problems that might be caused by CheR and CheB binding interactions, the NWETF segment of Tsr1 was excised and replaced by the linker. We expected that a single NWETF pentapeptide at the end of the Tsr2 subunit would be sufficient for function because these CheR/CheB tethers are known to function in *trans* [33–35]. In all single-chain constructs, hereafter designated Tsr1~Tsr2, the coding segment for the Tsr2 subunit was essentially a full-length *tsr* gene, beginning either at the first or second codon. The *tsr2* coding segment was spliced, using oligonucleotides encoding various polypeptide linkers, to a *tsr1* gene lacking some C-terminal *tsr* codons, including those for the NWETF segment.

Three basic Tsr1 lengths were tested: 545 or 546 amino acids (removing the NWETF pentapeptide), 517 amino acids (removing NWETF and all non-conserved C-terminal residues), and 533 residues (removing NWETF and roughly half of the non-conserved C-terminal amino acids) [36], a length similar to that of the low-abundance chemoreceptors Tap (dipeptide-sensing) and Trg (ribose and galactose-sensing). All Tsr1 constructs began with the first residue of Tsr, but some (designated 1*-) also carried a single amino acid insertion, created by the cloning process, between the second and third residues of Tsr. The minor differences in the N-terminal residues of Tsr1 or Tsr2 in some of the constructs proved irrelevant to single-chain receptor function, so for simplicity we will designate specific single-chain molecules by their Tsr1 and Tsr2 C-terminal residue numbers and by the intervening linker. For example, 545~551/L-1 denotes a single-chain molecule with a Tsr1 segment ending at residue 545 joined by a DPISSLAP amino acid linker to a Tsr2 segment ending at residue 551. Altogether, we created three basic Tsr1 lengths, six different linkers, and several different plasmid expression vectors for these constructs (Fig 1 and S1 Table). To avoid recombination between the tandemly duplicated *tsr* coding regions and consequent segregation of wild-type *tsr* genes, single-chain plasmids were generally characterized for function in recombination-deficient host strains.

### Optimized hosts for Tsr~Tsr function

We compared the ability of plasmids encoding wild-type and single-chain Tsr constructs in a strain lacking all MCP transducers (UU1448) to support serine chemotaxis on soft agar plates (S1A Fig). Wild-type Tsr expressed from plasmid pJC3 exhibited optimal function upon induction with 10–20 μM IPTG. At 10 μM IPTG, a pJC3-derived single-chain Tsr (545~551/L-1; plasmid pRR22) also supported serine chemotaxis, but the colonies expanded at only 40–50% of the wild-type Tsr rate. Tsr1~Tsr2 function declined at 20 μM IPTG, which indicates that the expression level of the single-chain molecules is not the rate-limiting factor in their chemotaxis performance.

We isolated two spontaneous variants of strain UU1448 that supported more robust chemotactic responses by single-chain Tsr constructs (see Methods and S1A Fig). Plasmid pRR22 in strain UU1449 functioned optimally at 5 μM IPTG, producing migration at about 45% of the wild-type rate. In strain UU1450, pRR22 functioned optimally at 10 μM IPTG induction, producing about 50% of the wild-type expansion rate. As anticipated from the behavior of pRR22 in UU1448, these relative improvements in performance are not simply due to increased expression of the single-chain construct, because Tsr1~Tsr2 function still declined at higher induction levels. Two other trivial explanations for the performance improvement can also be excluded. UU1449 and UU1450 remained recombination-deficient and, like UU1448, did not exhibit detectable proteolysis of Tsr1~Tsr2 molecules to Tsr-sized subunits (S1B Fig). Both strains also exhibited altered induction profiles for pJC3-mediated chemotaxis, indicating that wild-type, as well as single-chain, receptor molecules function more efficiently in UU1449 and UU1450. Although we have not determined the nature of the mutational change(s) in these strains, we suspect that they might alter folding efficiency or membrane insertion of the receptor molecules. UU1450 was chosen as the host for further studies of single-chain Tsr function.

### Effect of linker length on Tsr~Tsr function

To determine whether linker composition and/or length influenced Tsr1~Tsr2 performance, we made a series of single-chain constructs in parent plasmid pPM2, which carries an IPTG-inducible *tac* promoter regulated by a perfectly palindromic *lac* operator. [These single-chain constructs also contained a crosslinking reporter site (S366C) in the Tsr2 subunit for later trimer-based structural studies [6], which are not discussed in this report.] The “ideal” *lac* operator provides lower basal expression and more precise IPTG control of expression level [37, 38]. With this expression control system, wild-type Tsr exhibited optimal function at 75 μM IPTG, whereas the optimum inducer concentration for single-chain constructs was 50 μM IPTG. Accordingly, functional comparisons of single-chain to wild-type Tsr constructs were made at their respective optimal IPTG concentrations.

**Fig 2:**
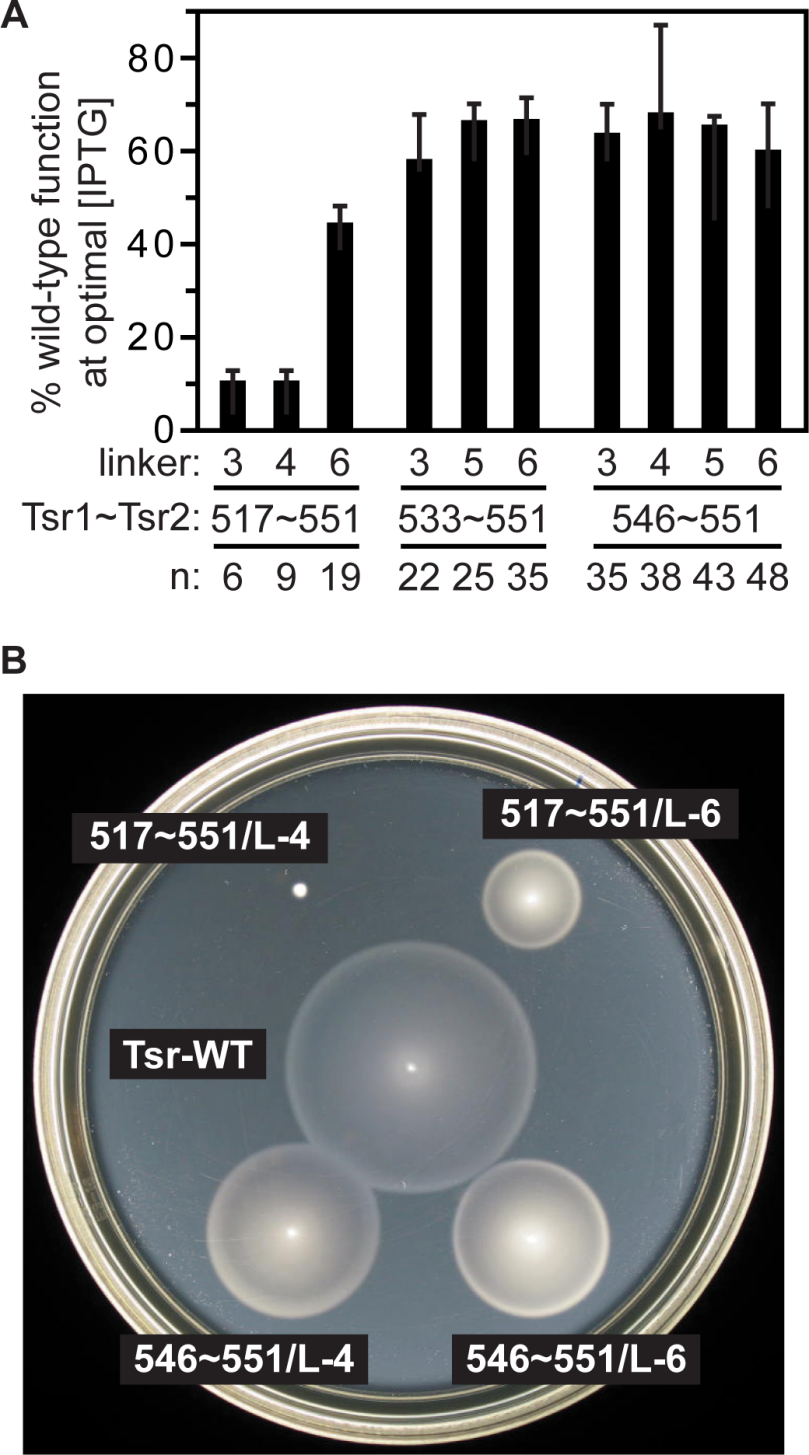
Effect of linker length and composition on single-chain receptor function. Tsr1~Tsr2 derivatives of plasmid pPM2 were tested for ability to mediate serine chemotaxis in host strain UU1450. Plasmids were (see S1 Table): pPM5 (517~551/L-3); pPM8 (517~551/L-4); pPM11 (517~551/L-6); pPM6 (533~551/L-3); pPM17 (533~551/L-5); pPM18 (553~551/L-6); pPM7 (546~551/L-3); pPM19 (546~551/L-4); pPM16 (546~551/L-5); pPM20 (546~551/L-6). All of these constructs carried the S366C reporter site in their Tsr2 subunit. (A) Colony sizes on tryptone soft agar produced by single-chain receptor strains (measured at 50 μM IPTG) relative to a wild-type Tsr control (pPM9 at 75 μM IPTG). Plates were incubated at 32.5°C for 7–8.5 hours. Error bars represent standard deviations of the average of three experiments. “n” = the number of amino acid residues between residue 517 of Tsr1 and the N-terminus of Tsr2. (B) Representative tryptone soft agar colony phenotypes at 75 μM IPTG.

Four 546~551 single-chain constructs with linkers of variable composition, ranging from six (L-3) to 19 (L-6) residues in length, exhibited comparable Tsr function, about 60–70% of the wild-type control (Fig 2). Removal of 13 additional Tsr residues (533~551 constructs) had no significant effect on function with the linkers tested. Removal of 16 more Tsr residues (517~551 constructs) supported approximately 45% function with the L-6 linker, but had essentially no function with the shorter L-3 or L-4 linkers, suggesting that linker length is more important to Tsr1~Tsr2 function than is linker composition. Thus, the overall length (designated “n” in Fig 2A) of the connection to Tsr2 following the last conserved residue in Tsr1 (517) seems to be the critical factor for functionality. Recall that wild-type Tsr has a 34-residue C-terminus following residue 517. Thus, Tsr~Tsr connections between 22 (533~551/L-3) and 48 (546~551/L-6) residues in length supported maximal single-chain function, a 19-residue connection (517~551/L-6) supported less function, and connection lengths of 9 (517~551/L-4) or fewer residues supported no function. Evidently, a too-short connection between Tsr1 and Tsr2 perturbs the native structure of single-chain receptors. The following sections explore the functional consequences of that structural change.

### Array formation by Tsr~Tsr molecules

Functional chemoreceptors typically form large signaling arrays at the cell pole(s) comprised of networked core complexes. We used a YFP-CheW fusion protein to ask whether nonfunctional single-chain receptors still formed cellular clusters. Wild-type Tsr (expressed from plasmid pPM9) made detectable clusters in 88% of UU1450 cells carrying pPA801, a YFP-CheW reporter plasmid. A functional single-chain receptor (546~551/L-4; expressed from plasmid pPM19) produced a comparable extent of cluster formation (84%). Similarly, a nonfunctional single-chain receptor (517~551/L-4; expressed from plasmid pPM8) produced detectable clusters in 84% of the cells examined. These results indicate that the structural defects of nonfunctional single-chain receptors do not prevent CheW binding and polar array formation.

### FRET analysis of Tsr-Tsr signaling responses

We used a FRET-based CheA kinase assay [25, 27] to examine intracellular signaling by the single-chain receptors in more direct fashion. This assay follows Förster resonance energy transfer *in vivo* between CheZ tagged with cyan fluorescent protein (CFP, the FRET donor) and CheY tagged with yellow fluorescent protein (YFP, the FRET acceptor). The FRET signal reflects the phosphorylation state of CheY, which in turn depends on the autophosphorylation rate of CheA, which is under receptor control. Attractant-induced changes in the FRET signal yield dose-response curves that provide measures of receptor stimulus sensitivity (*K*_*1/2*_) and signaling cooperativity (Hill coefficient) (see Methods).

**Fig 3:**
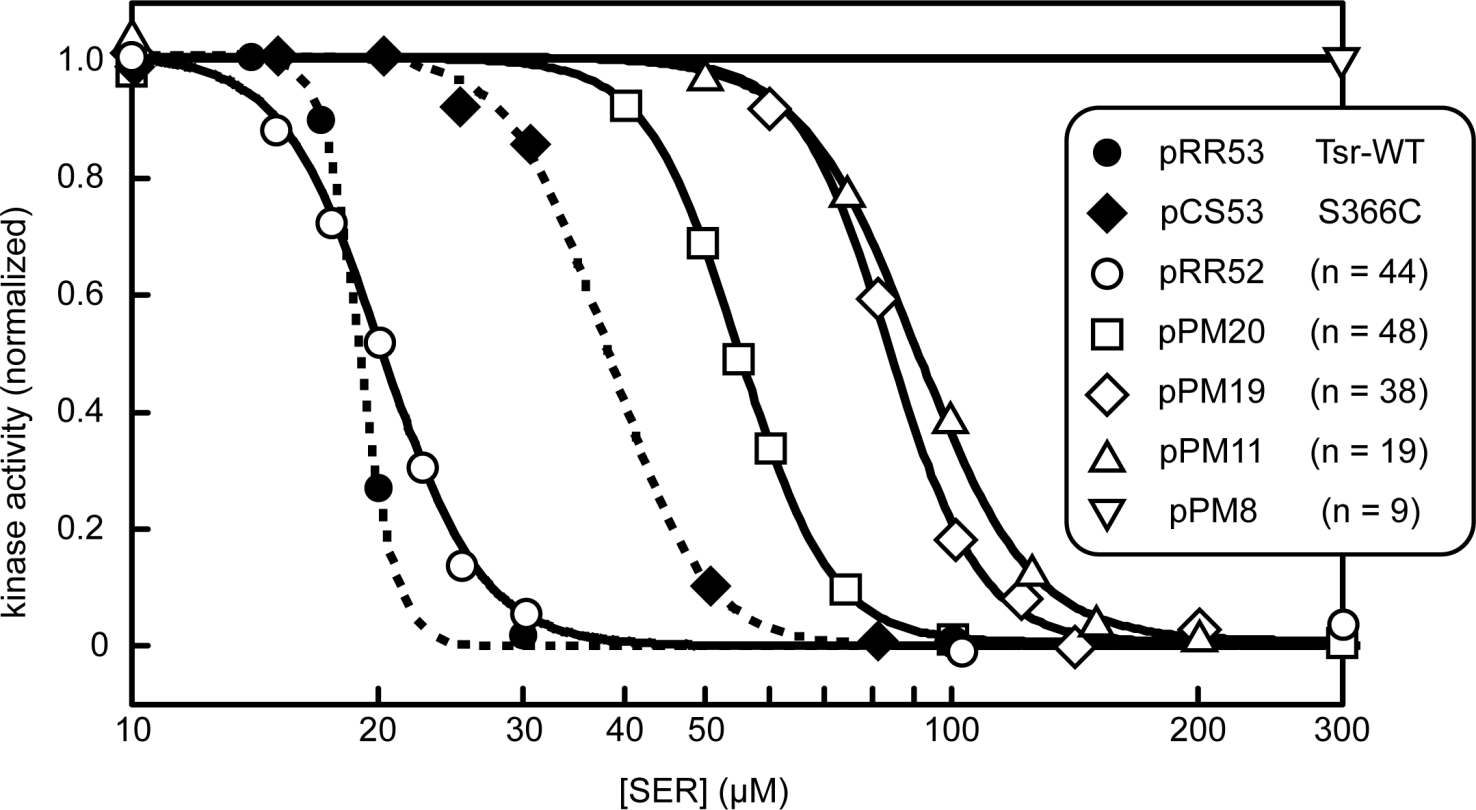
Dose-response properties of single-chain Tsr molecules. Plasmids expressing Tsr~Tsr constructs were tested for ability to down-regulate CheA kinase activity in response to serine stimuli in the FRET reporter strain UU2567. Fits of the FRET response data to a multi-site Hill equation are shown: solid lines = single-chain receptors; broken lines = wild-type control receptors. Number of residues (n) in the linking segments of single-chain receptors are indicated in parentheses. Receptor constructs, *K*_*1/2*_ concentrations and Hill coefficients were: pRR53 (Tsr-wt, *K*_*1/2*_ = 19 μM, Hill = 19); pCS53 [29] (Tsr-S366C, *K*_*1/2*_ = 37 μM, Hill = 7.6); pRR52 (545~551/L-2, *K*_*1/2*_ = 20 μM, Hill = 7.2); pPM20 (546~551/L-6, *K*_*1/2*_ = 55 μM, Hill = 7.8); pPM19 (546~551/L-4, *K*_*1/2*_ = 84 μM, Hill = 7.9); pPM11 (517~551/L-6, *K*_*1/2*_ = 91 μM, Hill = 6.2); pPM8 (517~551/L-4, high kinase activity, no serine response).

Plasmids encoding single-chain Tsr constructs were characterized in FRET reporter strain UU2567. This host lacks the CheR and CheB enzymes of the sensory adaptation system, thus avoiding any heterogeneity caused by modification of the receptor molecules. Under these conditions each Tsr subunit bears five adaptation sites: three glutamic acid residues (E304, E493, E502), representing unmethylated sites; and two glutamine residues (Q297 and Q311), which mimic methylated sites. Wild-type Tsr in the QEQEE modification state exhibited a *K*_*1/2*_ response to serine of 19 μM and a Hill coefficient of 19, indicative of a moderately sensitive, highly cooperative signaling response (Fig 3). A comparable single-chain construct (545~551/L-2, encoded by plasmid pRR52) produced a similarly sensitive response (*K*_*1/2*_ = 20 μM), but with reduced cooperativity (Hill = 7.2). The lower signaling cooperativity of the single-chain receptors could arise at the level of individual core complexes or through altered communication between core complexes in the receptor array and could account for the reduced chemotactic performance of single-chain receptors in soft agar assays.

Wild-type Tsr bearing the S366C trimer reporter site mediated slightly less sensitive (*K*_*1/2*_ = 37 μM) and less cooperative (Hill = 7.6) responses than fully wild-type Tsr molecules, owing most likely to some disulfide-bonded molecules in the S366C receptor population (Fig 3). Single-chain Tsr derivatives carrying the S366C site produced responses of comparable cooperativity, but reduced sensitivity. Their *K*_*1/2*_ values scaled inversely with the aggregate lengths of their connecting segment between Tsr1 and Tsr2: the longest linker (546~551/L-6) produced the most sensitive response (*K*_*1/2*_ = 55 μM); the shortest (517~551/L-4) failed to respond to serine, but evinced high kinase activity upon potassium cyanide challenge, which depletes cellular ATP, the phosphodonor for the CheA autophosphorylation reaction (see Methods). These findings parallel the soft agar and clustering phenotypes produced by the various single-chain receptors. All constructs, regardless of aggregate connection length, produced kinase-active core complexes and arrays. However, those with the longest connecting segments produced the most sensitive FRET responses and the most rapid colony expansions in soft agar.

### Crosslinking evidence for subunit-swapped Tsr~Tsr molecules

**Fig 4:**
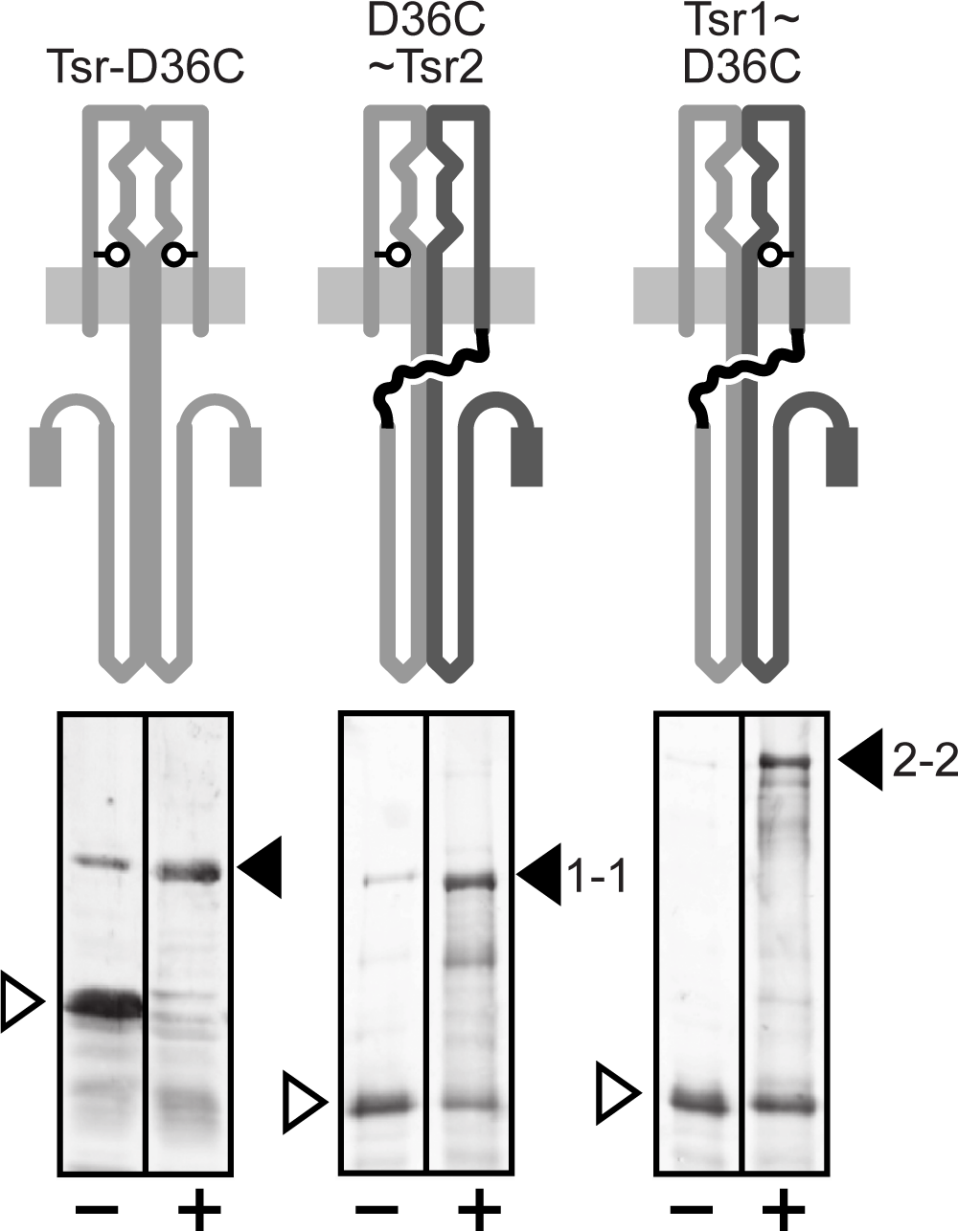
Detection of subunit-swapped single-chain molecules by disulfide crosslinking. Plasmid pPM9 and pPM13 derivatives (see S1 Table) encoding wild-type Tsr or single-chain (546~551/L-4) receptors with a D36C reporter site (white circles) in Tsr1 (pPM34) or Tsr2 (pPM33) were expressed in strain UU1609. Cell samples were treated with (+) or without (-) Cu-phenanthroline and analyzed by SDS-PAGE and anti-Tsr immunoblotting. Monomeric Tsr (first two lanes) was analyzed on a different gel from the single-chain constructs (lanes 3–6), owing to their substantial mobility differences. The different mobilities of the crosslinked single-chain products are due to the difference in the position of the disulfide bond within the crosslinked molecules. Uncrosslinked products (white arrows), crosslinked products (black arrows), “1–1” = disulfide between Tsr1 subunits; “2–2” = disulfide between Tsr2 subunits.

The soft agar phenotypes, clustering patterns, and FRET response properties of single-chain receptors indicated that they were promising subjects for investigating the functional architecture of trimer-based signaling complexes. However, we were concerned that single-chain receptor molecules might be able to exchange subunits, either during or after membrane insertion. Such swapping events would confound experimental applications of the single-chain molecules. To look for evidence of subunit swapping in single-chain receptor molecules, we employed a cysteine reporter site (D36C) at the dimer interface in the periplasmic domain [33] (Fig 4). Wild-type Tsr molecules with a D36C site in each subunit readily formed disulfide-bonded products in the presence of Cu-phenanthroline.

Single-chain Tsr molecules with a single D36C reporter in either Tsr1 or Tsr2 also formed crosslinked products upon Cu-phenanthroline treatment, indicative of swapping events that juxtapose two Tsr1 or two Tsr2 subunits (Fig 4). If single-chain subunits swap entirely at random, a maximum of 25% of the molecules should form same-subunit disulfides (Tsr1/Tsr1 or Tsr2/Tsr2, depending on the construct). However, after 10 minutes of treatment, approximately 50% of the reporter molecules had formed disulfides (S2 Fig). This proportion did not increase with longer treatment times and/or when new receptor synthesis was blocked. These findings are consistent with two alternative swapping scenarios that are not necessarily mutually exclusive: single-chain molecules could engage in repeated swapping interactions or exchanges could occur non-randomly with respect to the subunits involved.

### Functional evidence for subunit-swapped Tsr~Tsr molecules

To better assess the practical extent and functional consequences of subunit-swapping by single-chain receptor molecules, we asked if a Tsr subunit with a dominant lesion could disrupt the function of co-expressed single-chain molecules, as swapping scenarios might predict. In principle, Tsr molecules with a dominant lesion could block the function of co-expressed wild-type Tsr molecules in any of three different ways. First, they could spoil the function of trimers of receptor dimers that contain a mutant homodimer. Because receptors of different sensing types can form mixed trimers of dimers, trimer-spoiling Tsr mutants exert epistatic (mutant receptor phenotype is dominant over other MCPs in the trimer) or jamming (mutant receptor blocks stimulus response of the trimer unit) effects on heterologous receptors [21, 39]. Second, a Tsr with a dominant lesion could block function by competitively titrating shared signaling proteins, such as CheA and CheW. Competitive titrator mutants have not been explicitly demonstrated, but are expected to jam signaling by heterologous receptors. Third, Tsr molecules with a dominant negative defect would spoil the function of heterodimers that contain a mutant subunit. Thus, Tsr subunits with strictly dominant defects could only spoil the function of a single-chain receptor by forming hybrid molecules through subunit swapping.

**Fig 5:**
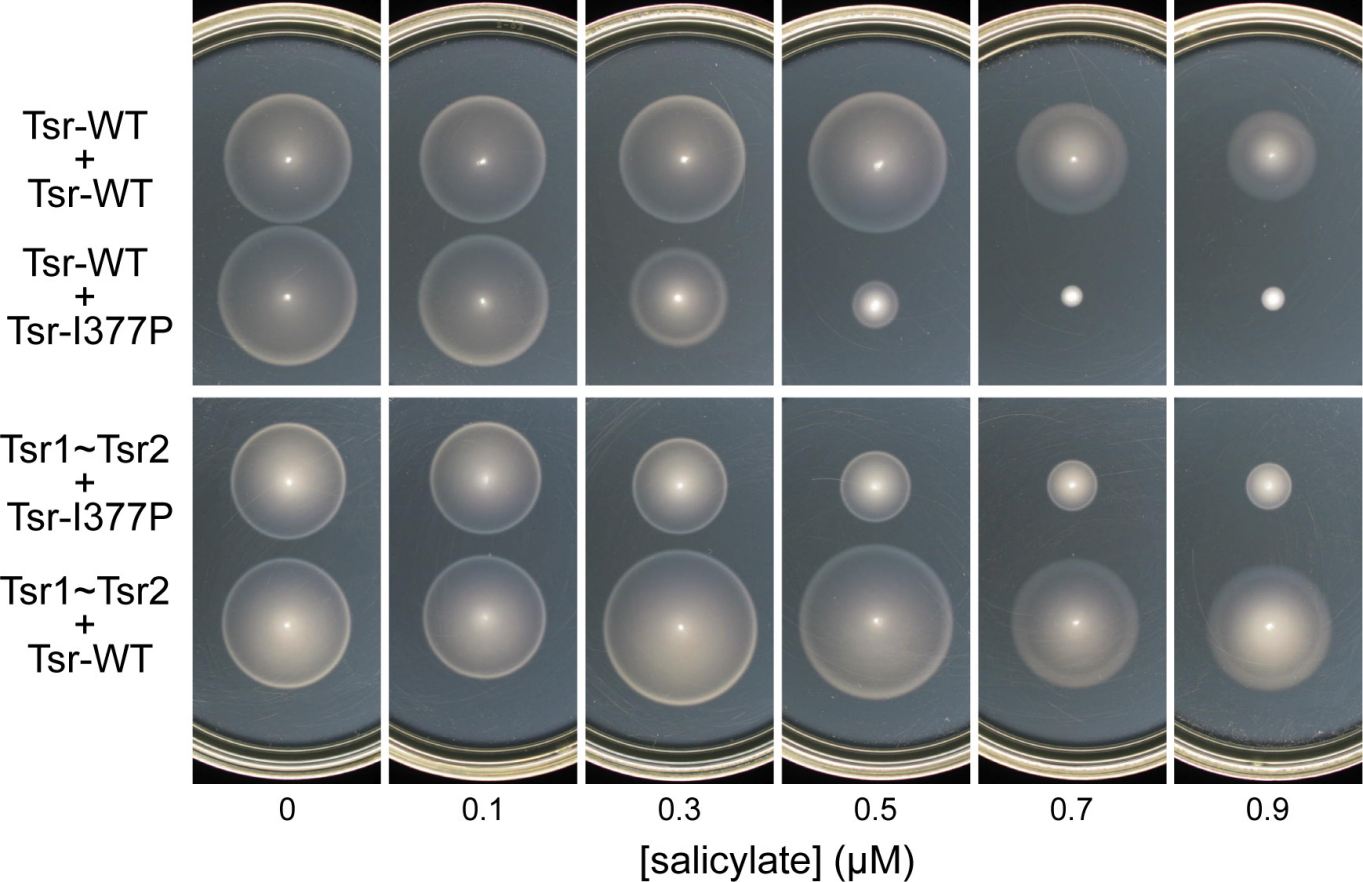
Spoiling of single-chain receptor function by dominant-negative Tsr subunits. Plasmids encoding various combinations of Tsr species (listed at left) were expressed in strain UU1450: Tsr-WT (pPM9 or pCS12 [7]); Tsr1~Tsr2 (pPM13 [546~551/L-4]); and Tsr-I377P (pCS12-I377P [7]). Cells were tested for chemotactic ability on tryptone soft agar plates. Results shown are representative of three independent experiments. Plates contained 50 μg ml^-1^ ampicillin, 12.5 μg ml^-1^ chloramphenicol, and different concentrations of sodium salicylate (for induction of pCS12 derivatives). Plates in the upper panels contained 75 μM IPTG (for induction of pPM9); plates in the lower panels contained 50 μM IPTG (for induction of pPM13).

To test for *in vivo* strand swapping with single-chain molecules, we chose a dominant Tsr defect (I377P) that prevents trimer formation and is, therefore, unable to jam the signaling of heterologous receptors [6, 7, 21, 40]. The I377P subunits effectively blocked Tsr function when co-expressed with wild-type subunits, completely abolishing function at roughly equimolar expression levels of wild-type and mutant subunits (Fig 5). The I377P subunits also reduced Tsr1~Tsr2 function, although the single-chain construct was less sensitive to the dominant effect. This experiment provides physiological evidence that single-chain molecules can undergo subunit-swapping interactions with monomeric Tsr subunits, but does not tell us whether one of the single-chain subunits, Tsr1 or Tsr2, is more prone to engage in swapping than the other and whether the displaced subunit remains unpaired.

### Effect of recessive lesions on Tsr~Tsr function

**Fig 6:**
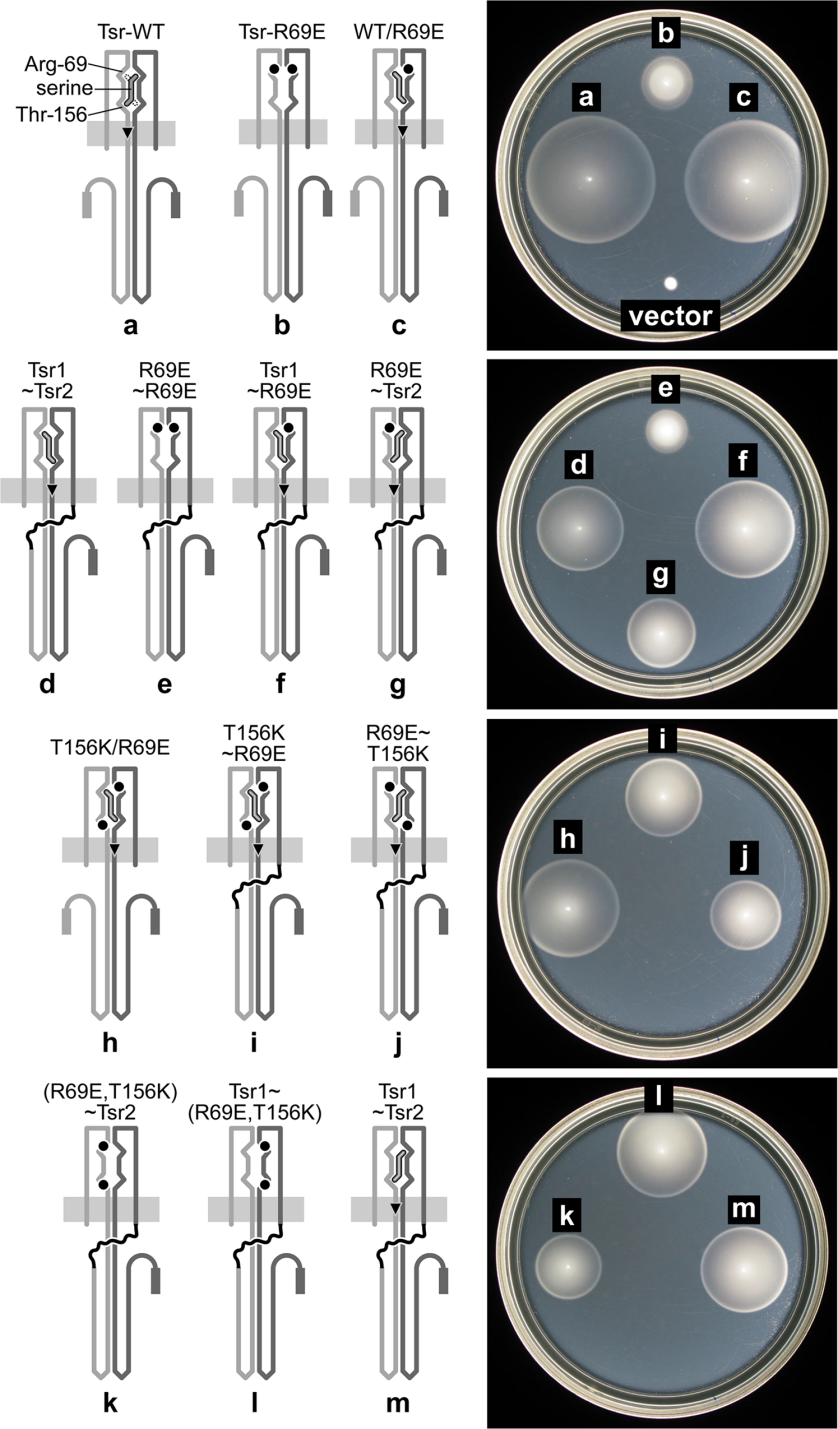
Function of single-chain Tsr molecules bearing recessive serine-binding defects. Plates show tryptone soft agar phenotypes of UU1450 colonies containing two compatible plasmids, expressing the Tsr receptors shown in the cartoons. (Representative results of five independent experiments.) All single-chain receptors (d-g,i-m) were expressed from pRR52 derivatives (chloramphenicol resistance; salicylate-inducible expression of Tsr1~Tsr2 [545~551/L-2]) in combination with a pCJ30 vector plasmid (ampicillin resistance) [48]. Monomeric Tsr subunits were expressed from pJC3 (ampicillin resistance; IPTG-inducible Tsr) in combination with pPA114 (chloramphenicol resistance; salicylate-inducible Tsr) [21] (a), pJC3-R69E in combination with control vector pKG110 (chloramphenicol resistance) (b), pJC3-R69E in combination with pPA114 (c), or pJC3-R69E in combination with pPA114-T156K (h). The vector control was pCJ30 (ampicillin resistance) in combination with pKG110. Binding site lesions are depicted by black circles on the cartoons and noted in the labels above each cartoon. For example, (R69E, T156K)~Tsr2 denotes Tsr1~Tsr2 with R69E and T156K lesions in Tsr1 and a wild-type Tsr2. Note: Even though there are two potential binding sites, only one serine molecule can bind a Tsr homodimer at a time. Slashes (e.g., WT/R69E) denote heterodimers produced by co-expression of monomeric Tsr subunits with the indicated mutations. Molecules capable of binding serine are depicted with ligand and a black triangle indicating the subunit that transmits the stimulus. Plates contained 15 μM IPTG and 0.6 μM Na salicylate.

To determine whether the Tsr1 and Tsr2 components of single-chain receptor molecules contribute equally to signaling, we compared the function of Tsr~Tsr constructs that had a recessive lesion in one or the other subunit. The Tsr periplasmic sensing domain possesses two symmetric ligand-binding sites at the dimer interface [41], but, owing to high negative cooperativity, only one molecule of attractant effectively binds per dimer. Arginine-69 in one subunit and threonine-156 in the other are key determinants of the serine-binding site [41–45]. The subunit in which T156 participates in ligand binding is the one that transmits the CheA-deactivating piston motion to the signaling domain [46, 47] (Fig 6A). Thus, Tsr homodimers with the R69E binding site lesion in both subunits (Fig 6B) cannot bind ligand, but heterodimers with one wild-type and one R69E mutant subunit can bind serine in one orientation and transmit a transmembrane signal through the R69E subunit (Fig 6C). Accordingly, we constructed a series of single-chain derivatives with the R69E binding site lesion in various configurations (Fig 6E–6G) and assessed their signaling properties with soft agar assays and with FRET kinase assays (Table 1).

**Table 1:** FRET kinase assays of Tsr~Tsr molecules with serine binding-site lesions.

Tsr~Tsr variant^*a*^	Fig 6 schematic	Expression plasmid	K_1/2_ μM serine	Hill coefficient
Tsr1~Tsr2	d & m	pRR52	20	7.2
Tsr1~R69E	f	pRR58	55	5.9
R69E~Tsr2	g	pRR68	99	3.9
T156K~R69E	i	pRR67	NR-ON^*b*^	NR-ON
R69E~T156K	j	pRR65	NR-ON	NR-ON
(R69E,T156K)~Tsr2	k	pRR71	NR-ON	NR-ON
Tsr1~(R69E,T156K)	l	pRR60	353	2.0
R69E~R69E	e	pRR64	3800	9.0

^*a*^ Tsr~Tsr variants were derivatives of Tsr1(1–545)~Tsr2(1–551)/L-2 encoded by plasmid pRR52. UU2567 was the host strain for these FRET experiments.

^*b*^ NR-ON: little or no response to 10 mM serine; high kinase activity.

Single-chain molecules with the R69E lesion in either Tsr2 (Fig 6F) or Tsr1 (Fig 6G) supported chemotaxis in soft agar and produced similar serine responses in FRET assays (Table 1). Conversely, the R69E lesion in both Tsr1 and Tsr2 greatly diminished chemotaxis function (Fig 6E) and greatly elevated the serine response threshold in the FRET assay (Table 1). These findings indicate that both Tsr1 and Tsr2 can transmit a ligand-binding signal, but they may not be equally proficient at doing so. The single-chain construct with R69E in Tsr2 (*i*.*e*., Tsr1~Tsr2-R69E; Fig 6F) generally produced faster colony expansion in soft agar than did the construct with R69E in Tsr1 (Tsr1-R69E~Tsr2; Fig 6G), suggesting that ligand-binding signals might travel more effectively through the Tsr2 subunit than through the Tsr1 subunit. The FRET assays revealed a similar subunit asymmetry: the single-chain receptor with a nonmutant Tsr1 subunit (Fig 6F) had slightly superior response parameters than its counterpart with a nonmutant Tsr2 subunit (Fig 6G) (*K*_*1/2*_ = 55 *vs*. 99 μM; Hill coefficient = 5.9 *vs*. 3.9; Table 1).

To further investigate the basis for signaling asymmetry in single-chain Tsr molecules, we constructed Tsr1~Tsr2 derivatives with the T156K lesion in one subunit and the R69E lesion in the other. These *trans* constructs (Tsr1-T156K~Tsr2-R69E and Tsr1-R69E~Tsr2-T156K) functioned in the chemotaxis assay (Fig 6I and 6J), indicating that Tsr1 and Tsr2 can work together to sense and transmit ligand-binding information. There was no evident difference in their chemotaxis proficiency in the soft agar assay, demonstrating that Tsr1 and Tsr2 can transmit comparable ligand-binding signals when constrained to do so. In contrast, in the FRET assay both *trans* constructs produced high kinase activity, but were non-responsive to serine (Table 1). This performance difference might reflect the fact that the FRET assay monitors fewer than a thousand cells, whereas soft agar plates sample millions of cells, only a small fraction of which need to be chemotaxis-proficient to produce a positive colony morphology.

The *cis* control experiments for these single-chain variants (Tsr1-R69E/T156K~Tsr2 and Tsr1-R69E/T156K~Tsr2) provided some direct evidence for subunit swapping events. Single-chain molecules with R69E and T156K in the same subunit, either Tsr1 or Tsr2, should not be able to bind ligand, yet such constructs mediated chemotactic behavior comparable to the *trans* constructs on soft agar plates (Fig 6K and 6L). In the *cis* arrangements, the single-chain receptor with a nonmutant Tsr1 subunit proved more effective, both in the soft agar assay (Fig 6K vs. 6L) and in the FRET assay (*K*_*1/2*_ = 353 μM *vs*. locked-on; Table 1). We suggest that these signaling properties arise from subunit-swapping interactions that allow single-chain receptors to form hybrid molecules with functional ligand-binding sites. In the FRET assays, receptors with dual lesions in a *cis* arrangement mediated, at best, rather insensitive (*K*_*1/2*_ = 353 μM) responses compared to a single-chain molecule with no binding site lesions (*K*_*1/2*_ = 20 μM) (Table 1), so subunit swapping does not seem to be a major contributor to the signaling properties of single-chain receptors, at least in FRET-based kinase assays.

## Discussion

This study demonstrates that single-chain versions of the *E*. *coli* serine chemoreceptor are capable of stimulus detection, transmembrane signaling, and kinase control responses that are nearly comparable to those of native Tsr homodimers. Two factors identified in this initial work, the length of the polypeptide joining the Tsr1 and Tsr2 subunits and strand-swapping interactions between molecules, influenced single-chain receptor function and will need to be taken into account in subsequent experimental applications of single-chain receptors.

### Functional role of the linker in single-chain receptor molecules

The length of the polypeptide linker joining the two subunits of a single-chain receptor was critical to function; its amino acid composition was not. Tsr1 subunits ending at residue 517, the last conserved C-terminal position in the receptor kinase control domain, supported single-chain function with a 19-residue linker segment, but not with shorter linkers of six or nine residues.

**Fig 7:**
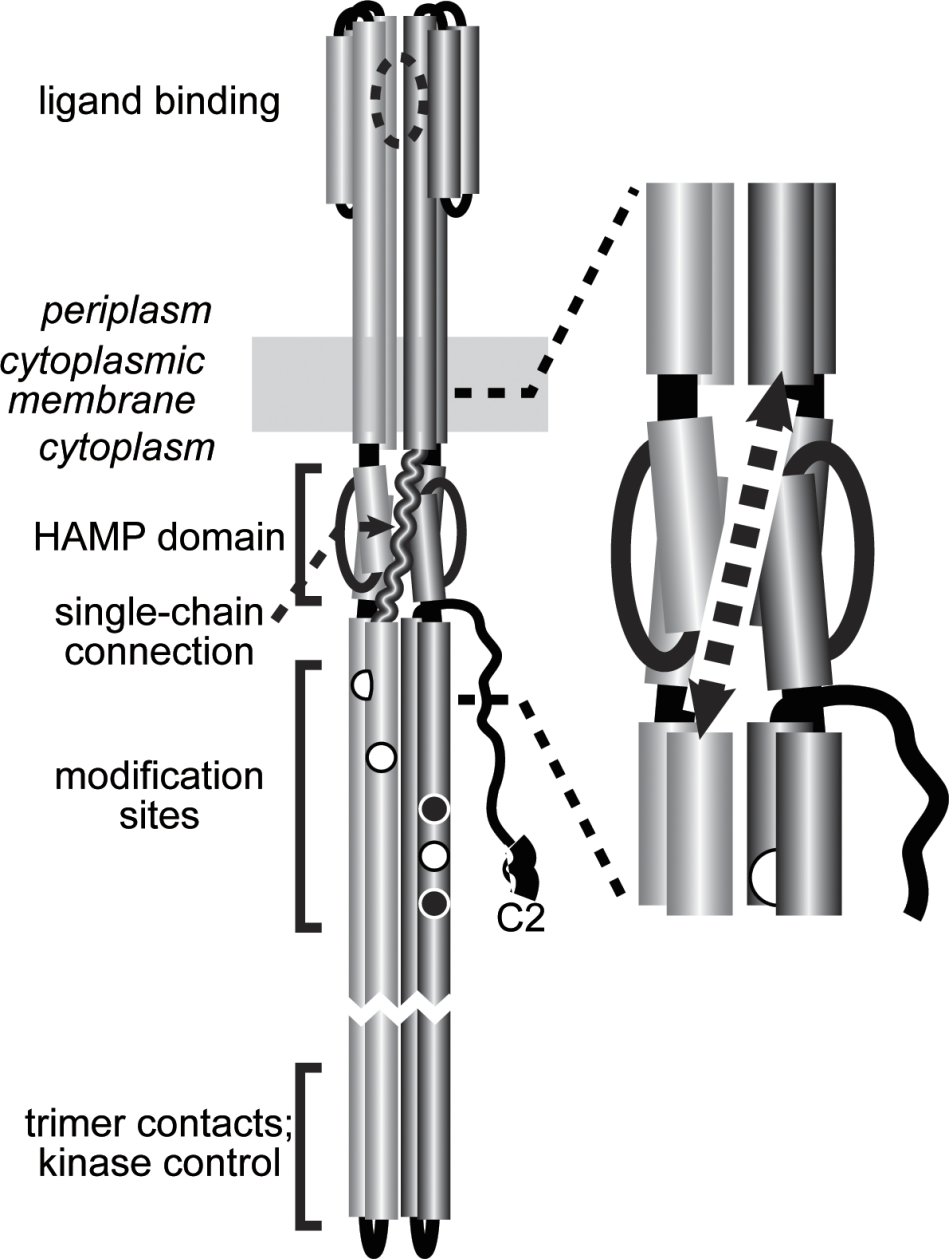
Functional architecture of single-chain MCP molecules. Cartoon of an MCP homodimer with one subunit shaded light gray, the other shaded dark gray. Cylindrical segments represent alpha-helices, drawn approximately to scale. The wavy segment (dashed arrow on the right) is the polypeptide connection linking the two subunits in a single-chain receptor molecule. An expanded view of the transmembrane-HAMP region and the connecting polypeptide is shown on the right.

The signaling defects of single-chain receptors with too-short linkers shed new light on mechanistic aspects of receptor signaling. Such receptors (*e*.*g*., 517~551/L-4; Fig 2 and Fig 3) failed to support chemotaxis in soft agar assays, but made kinase-active core complexes and arrays. In FRET assays, their kinase activity could not be inhibited by high levels of serine, indicative of locked-on output. In principle, locked-on behavior could arise from a block at the stimulus detection or transmembrane signaling step, but in this case it might be due to a structural perturbation of the HAMP (Histidine kinases, Adenylate cyclases, Methyl accepting proteins and Phosphatases) domain, which relays transmembrane signals to the kinase-control tip of the receptor molecule [49]. The linker segment in a single-chain receptor traverses the HAMP domain (Fig 7) and, if too short, might constrain or distort the 4-helix HAMP bundle. The helices of the native HAMP bundle extend over six turns, a length of about 32 Å. Assuming 3 Å per residue for an extended polypeptide, the linker segment would need to be at least 11–12 residues in length to bridge the shortest distance across the HAMP domain. If the linker crosses HAMP at an angle, as shown in Fig 7, the minimal linker length would be even longer. Thus, a linker of 6–9 residues should not be long enough to accommodate a native HAMP bundle. Ablation or destabilization of the HAMP domain is known to produce locked-on behavior [50, 51], consistent with the signaling defect of single-chain receptors with too-short linkers.

A 19-residue linker in the 517~551 single-chain molecule evidently provides ample freedom of motion for the HAMP domain to transmit attractant-induced kinase-off signals to the output domain of the receptor. Several models have been proposed for the kinase-on and kinase-off conformations of the HAMP domain in chemoreceptors, ranging from changes in bundle packing [52] or dynamics [53] to more dramatic shifts between compact and expanded structures [54]. It seems unlikely that the linker segments in functional single-chain receptors could accommodate large-scale changes in HAMP domain volume, but it could be possible to more precisely determine the structural limits of HAMP domain signaling changes with a series of single-chain receptors that have one-residue increments in linker length over the critical range of 10–20 residues.

### Functional role of the NWETF pentapeptide in single-chain receptor molecules

Efficient chemotactic signaling by a single-chain receptor would most likely require that some molecules in the receptor ensemble bear the C-terminal NWETF pentapeptide. This segment interacts with CheR and CheB molecules [31, 32], thereby enhancing the receptor methylation and demethylation reactions, which are needed for gradient sensing and sensory adaptation. In single-chain molecules only the Tsr2 subunit carries the pentapeptide, but these subunits probably would not need to reside in molecules capable of sensing or signaling because in a receptor array the CheR/CheB tethering function can be supplied in *trans* by NWETF segments on non-signaling receptors [33–35]. Receptors with two NWETF-bearing subunits can assist the adaptation of 5–7 neighboring receptors [55]. The effective neighborhood size might be somewhat smaller for Tsr1~Tsr2 molecules that have a single NWETF subunit [56], but it seems unlikely that this factor would limit the functionality of single-chain receptors. Indeed, single-chain receptors with locked-on outputs in a host lacking the CheR and CheB enzymes (see Table 1) mediated substantial chemotactic responses in an adaptation-proficient host (see Fig 6), indicating that the signaling properties of these single-chain receptors are subject to sensory adaptation control.

### Functional role of strand swapping in single-chain receptor molecules

**Fig 8:**
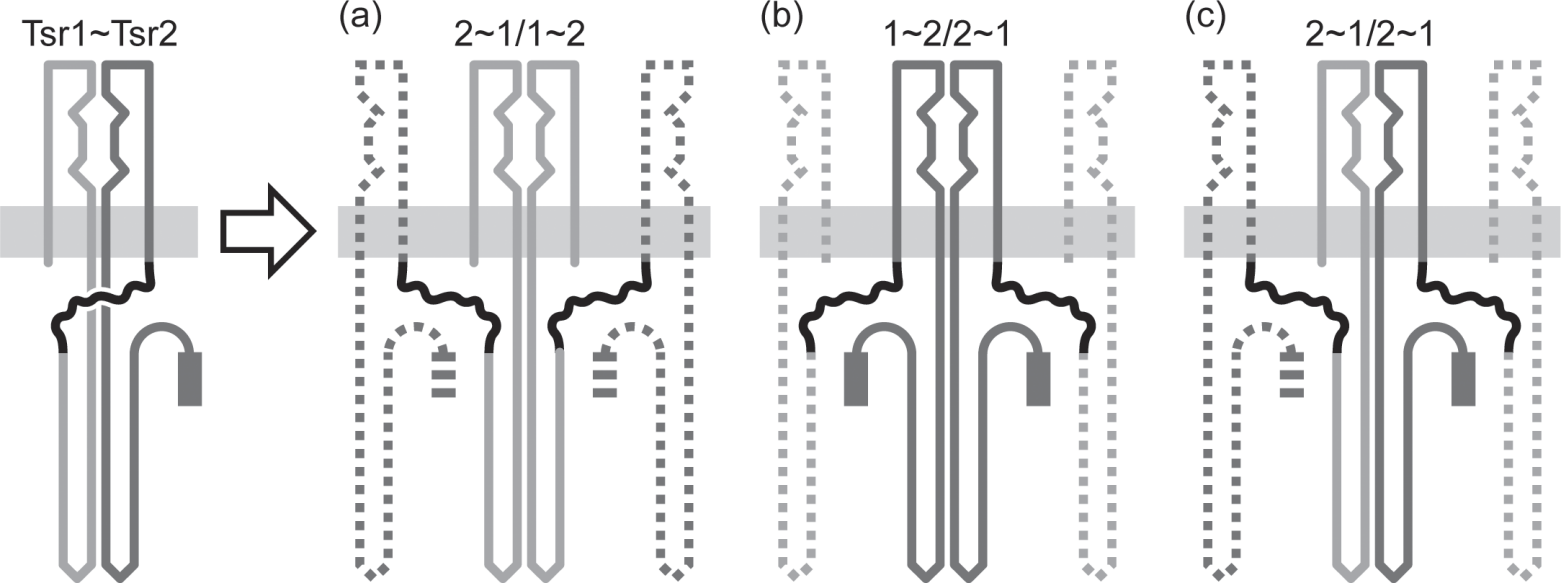
Subunit-swapping scenarios in single-chain MCP molecules. The Tsr1 subunit is light gray; the Tsr2 subunit is dark gray. Subunit exchanges between single-chain molecules could create paired Tsr1 subunits (a), paired Tsr2 subunits (b) or a Tsr1 subunit from one molecule paired with a Tsr2 subunit from the other (c). In all cases, the dashed segments indicate unpaired subunits resulting from the initial swap. Their possible fates are discussed in the text.

We detected strand-swapping interactions between single-chain receptor molecules with a disulfide trapping assay. Single-chain molecules that carried a single cysteine residue in the periplasmic domain of either Tsr1 or Tsr2 formed crosslinked products under oxidizing conditions. These results provide direct evidence for swapped molecules with Tsr1-Tsr1 and Tsr2-Tsr2 interfaces and imply that Tsr1-Tsr2 interfaces probably also exist in single-chain populations (Fig 8).

Single-chain receptors with dual recessive serine-binding lesions in one subunit indicated that swapped molecules with a Tsr1/Tsr1 interface (Fig 8A) supported faster chemotactic expansions and more sensitive serine responses than did swapped molecules with a Tsr2/Tsr2 interface (Fig 8B). Perhaps the presence of a linker segment at the N-terminus of the Tsr2 subunit impairs its folding or membrane insertion or signaling properties in swapped molecules.

Receptor trimers assembled in the presence of CheA and CheW exhibit few exchanges with the pool of newly made receptor dimers [7]. Subunit swaps may be less common under these conditions, but they nevertheless occur. We found, for example, that high levels of monomeric receptor subunits bearing a dominant negative functional lesion spoiled the function of single-chain receptor molecules, presumably through strand-swapping interactions. Moreover, single-chain molecules bearing complementary binding-site lesions in a *cis* arrangement, which must form swapped species in order to bind ligand, exhibited serine responses both in soft agar and FRET assays. However, the FRET response was an order of magnitude less sensitive than for single-chain receptor constructs capable of detecting serine without required subunit-swapping events. These findings indicate that in the presence of their ternary complex partners, CheA and CheW, single-chain receptors may not participate in extensive subunit swapping behavior.

The first receptor subunit of a single-chain molecule should be available for dimerizing interactions immediately upon insertion into the membrane, most likely before synthesis and insertion of the second subunit are complete. Accordingly, swapping events between single-chain molecules should lead to transiently unpartnered subunits, which would most likely be substrates for proteolysis. Yet we observed few monomer-sized Tsr proteins in functional single-chain receptor populations, implying either that swapping events occur infrequently under those conditions or that they are mainly reciprocal in nature. Reciprocal swapping interactions between single-chain molecules could impose additional structural constraints at the level of individual core signaling complexes and/or the networked receptor array. For example, swapping could create satellite dimers physically connected to the trimers of dimers in a core signaling complex. Such appendages could influence the signaling properties of the core complex and might also perturb the higher-order geometry of core complexes in the array. Alternatively, a swapping event between single-chain members of a trimer could trigger reciprocal swaps that physically interconnect the individual receptor molecules of a core signaling complex.

Extensive subunit swapping has not been reported in two other examples of engineered single-chain proteins in *E*. *coli*. Takatsuka and Nikaido [57] constructed a "giant gene" encoding three covalently connected subunits of AcrB, a component of a multidrug efflux pump, to show that AcrB operated as a trimer. A lesion in any one subunit of the single-chain AcrB trimer destroyed its ability to expel antibiotics from the cell, arguing that all three subunits of the covalent trimer were critical for export function. While they did not directly assess swapping, a single-chain AcrB dimer failed to confer full antibiotic resistance, implying that subunit swapping and proteolytic trimming did not create a fully functional AcrB trimer. More recently, Greenswag et al. [58] constructed and characterized soluble single-chain molecules containing two kinase control subunits of the aspartate chemoreceptor Tar. The majority created dimers and not larger aggregates from swapped domains. Some of those proteins exhibited minor levels of higher molecular weight "aggregates" that could represent swapped molecules.

### Naturally occurring and synthetic single-chain receptors

The genomes of *Rhodospirillum centenum* [16] and a handful of other microbes [17] encode soluble single-chain versions of MCP signaling domains similar to the Tar constructs described above [58]. The functional roles of these proteins and whether they undergo subunit swapping interactions are unknown. Subunit swapping, like that observed with our full-length single-chain receptors, could conceivably be essential for proper function of soluble single-chain receptor signaling domains. For example, subunit interlacing might facilitate signaling or enable the soluble molecules to form a signaling lattice. Conversely, these natural single-chain molecules may have evolved mechanisms to avoid swapping subunits, which, if identified, might be applicable to the sorts of synthetic single-chain receptor molecules described in this report.

We constructed single-chain molecules of the serine chemoreceptor as a means to manipulate subunit composition and arrangements in receptor trimers of dimers, which are important architectural and functional components of chemoreceptor core complexes and signaling arrays. This report describes the first step toward that goal. Although single-chain receptors can support chemotactic signaling, strand-swapping interactions between the covalently connected receptor subunits could conceivably limit their utility. Clearly, we need to learn more about the cellular conditions that foster swapping events, the extent to which such events occur in single-chain receptor populations, and the molecular nature of the swapped products.

## Supporting Information

S1 Fig Properties of wild-type and single-chain Tsr molecules in different host strains. (A) Plasmids pJC3 (Tsr-WT) and pRR22 (Tsr1~Tsr2; 545~551/L-1) were introduced into strains UU1448 and derivatives UU1449 and UU1450. Transformant colonies were tested on soft agar plates containing 50 μg ml^-1^ ampicillin and different concentrations of IPTG and incubated at 32.5°C for 8 hours. (B) Protein expression from pJC3 and pRR22 as analyzed by Western blot with anti-Tsr serum.
(TIF)

S2 Fig Linear range of cross-linked formation of single-chain molecules. UU1609 carrying pPM33 (546~551/L-4 Tsr1~Tsr2-D36C) was grown at 30°C in tryptone broth containing 50 μg ml^-1^ ampicillin and 10 μM IPTG. Upon reaching OD_600_ = 0.5, portions of the cultures were treated with 500 μg ml^-1^ chloramphenicol and incubated at 30°C with shaking for an additional 90 minutes. The untreated (A) and chloramphenicol-treated (B) samples were washed, resuspended with or without chloramphenicol, and incubated with Cu-phenanthroline at 37°C. Samples were removed at the indicated times, the reaction stopped with 10mM NEM and 10mM EDTA, and the samples were analyzed for crosslinked products by SDS-containing polyacrylamide gels. The data points are averages of two experiments. Error bars represent standard deviations.
(TIF)

S1 Table Plasmids. (DOCX)
