# Supplementary figures and images for: Chemotactic Signaling by Single-Chain Chemoreceptors
Source: PLoS One. 2015 Dec 28;10(12):e0145267. doi: 10.1371/journal.pone.0145267 (PMC4692393; doi:10.1371/journal.pone.0145267)

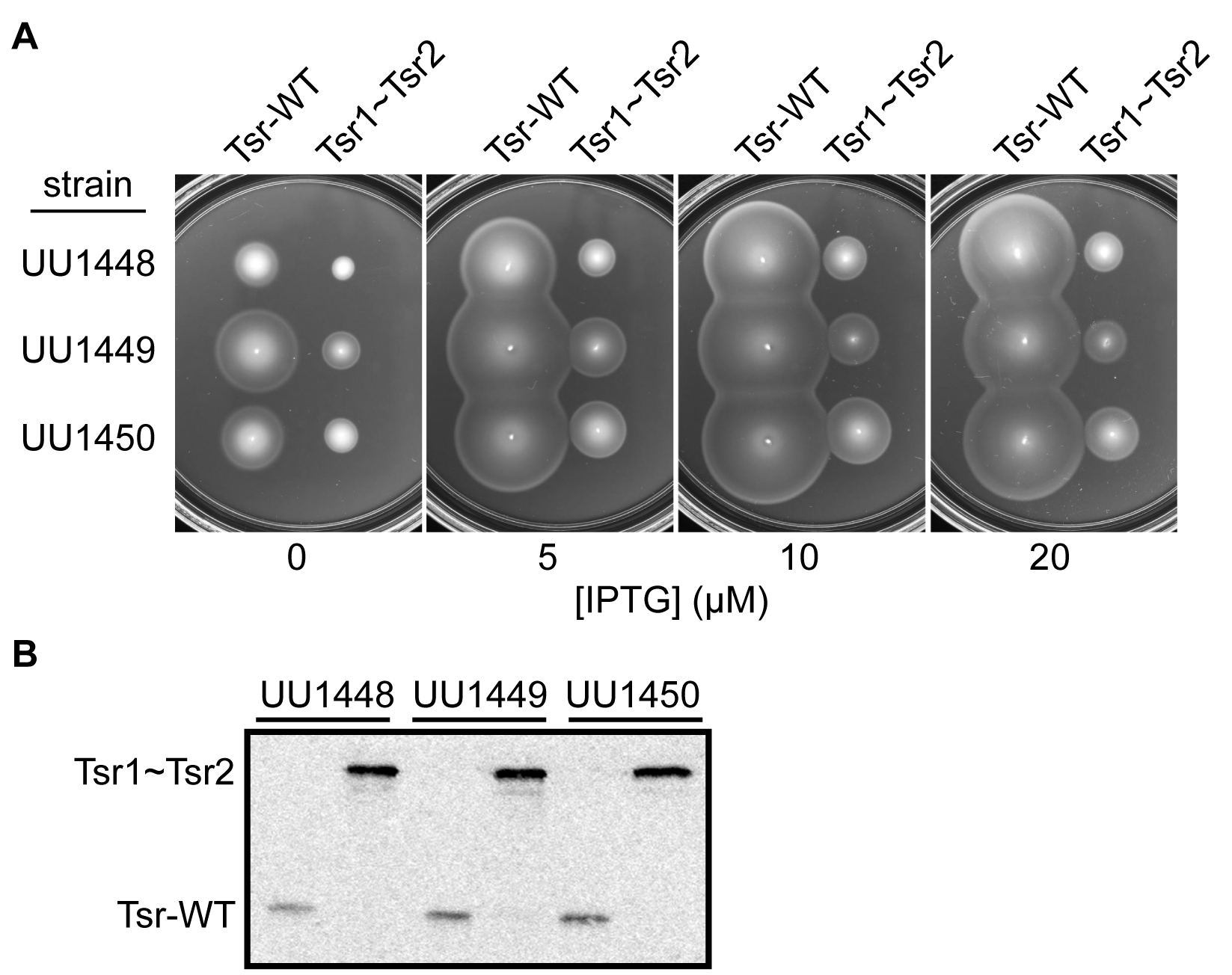

Supplement: S1 Fig — (A) Plasmids pJC3 (Tsr-WT) and pRR22 (Tsr1~Tsr2; 545~551/L-1) were introduced into strains UU1448 and derivatives UU1449 and UU1450. Transformant colonies were tested on soft agar plates containing 50 μg ml-1 ampicillin and different concentrations of IPTG and incubated at 32.5°C for 8 hours. (B) Protein expression from pJC3 and pRR22 as analyzed by Western blot with anti-Tsr serum. (TIF) [file pone.0145267.s001.tif]

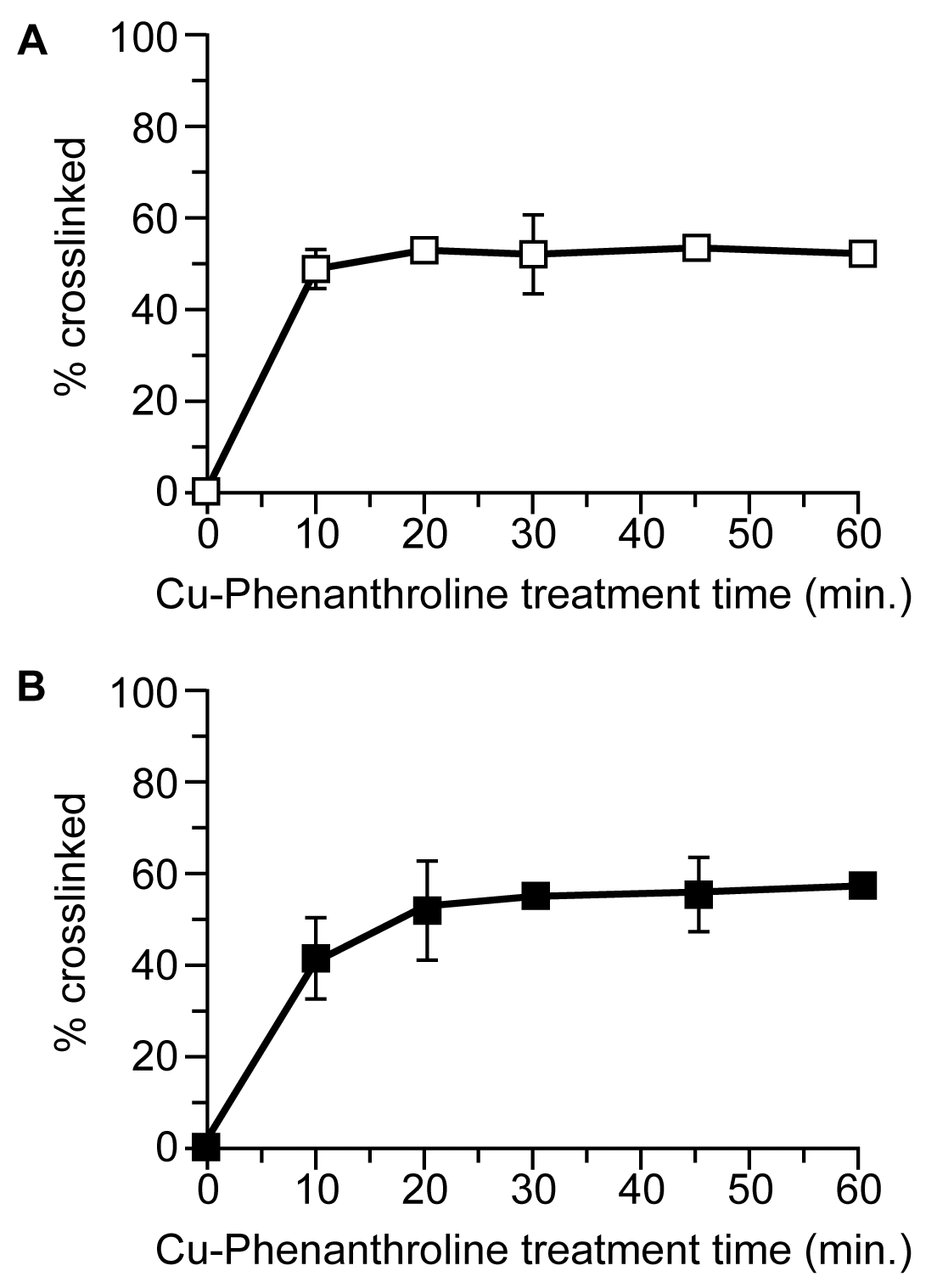

Supplement: S2 Fig — UU1609 carrying pPM33 (546~551/L-4 Tsr1~Tsr2-D36C) was grown at 30°C in tryptone broth containing 50 μg ml-1 ampicillin and 10 μM IPTG. Upon reaching OD600 = 0.5, portions of the cultures were treated with 500 μg ml-1 chloramphenicol and incubated at 30°C with shaking for an additional 90 minutes. The untreated (A) and chloramphenicol-treated (B) samples were washed, resuspended with or without chloramphenicol, and incubated with Cu-phenanthroline at 37°C. Samples were removed at the indicated times, the reaction stopped with 10mM NEM and 10mM EDTA, and the samples were analyzed for crosslinked products by SDS-containing polyacrylamide gels. The data points are averages of two experiments. Error bars represent standard deviations. (TIF) [file pone.0145267.s002.tif]
