# Supplementary material for: Chemotactic Signaling by Single-Chain Chemoreceptors
Source: PLoS One. 2015 Dec 28;10(12):e0145267. doi: 10.1371/journal.pone.0145267 (PMC4692393; doi:10.1371/journal.pone.0145267)
Supplement: S1 Table — (DOCX) [file pone.0145267.s003.docx]

| **Plasmid** | **Relevant features ^a^** | **Reference** |
| --- | --- | --- |
| pCJ30 | *bla lacI* *P_tac_* *lacO* | [48] |
| pCS12 | *cat* *nahR P_nahG_* [*tsr*(1-551)] | [7] |
| pCS12-I377P | pCS12 [*tsr*(1-551/I377P)] | [7] |
| pCS53 | pRR53 [*tsr*(1-551)/S366C] | [29] |
| pJC3 | pCJ30 [*tsr*(1-551)] | [21] |
| pJC3-R69E | pJC3 [*tsr*(1-551/R69E)] | this work |
| pJC3-T156K | pJC3 [*tsr*(1-551/T156K)] | this work |
| pKG110 | *cat* *nahR P_nahG_* [NdeI…NotI…KpnI] | this work |
| pPA114 | *cat* *nahR P_nahG_* [*tsr*(1-551)] | [21] |
| pPA801 | c*at P_ara_* [*yfp-cheW*] | [29] |
| pPM2 | *bla* *P_tac_ lacO_ideal_* [PstI…EcoRI…NheI…SalI] ^b^ | this work |
| pPM5 | pPM2 [*tsr*(1*-517) (L-3) *tsr*(2-551/S366C)] ^c^ | this work |
| pPM6 | pPM2 [*tsr*(1*-533) (L-3) *tsr*(2-551/S366C)] ^c^ | this work |
| pPM7 | pPM2 [*tsr*(1*-546) (L-3) *tsr*(2-551/S366C)] ^c^ | this work |
| pPM8 | pPM2 [*tsr*(1*-517) (L-4) *tsr*(2-551/S366C)] ^c^ | this work |
| pPM9 | pPM2 [*tsr*(1*-551)] [PstI…SalI] ^c^ | this work |
| pPM9-D36C | pPM9 [*tsr*(1*-551/D36C)] ^c^ | this work |
| pPM11 | pPM2 [*tsr*(1*-517) (L-6) *tsr*(2-551/S366C)] ^c^ | this work |
| pPM13 | pPM2 [*tsr*(1*-546) (L-4) *tsr*(2-551)] ^c^ | this work |
| pPM16 | pPM2 [*tsr*(1*-546) (L-5) *tsr*(2-551/S366C)] ^c^ | this work |
| pPM17 | pPM2 [*tsr*(1*-533) (L-5) *tsr*(2-551/S366C)] ^c^ | this work |
| pPM18 | pPM2 [*tsr*(1*-533) (L-6) *tsr*(2-551/S366C)] ^c^ | this work |
| pPM19 | pPM2 [*tsr*(1*-546) (L-4) *tsr*(2-551/S366C)] ^c^ | this work |
| pPM20 | pPM2 [*tsr*(1*-546) (L-6) *tsr*(2-551/S366C)] ^c^ | this work |
| pPM33 | pPM2 [*tsr*(1*-546) (L-4) *tsr*(2-551/D36C)] ^c^ | this work |
| pPM34 | pPM2 [*tsr*(1*-546/D36C) (L-4) *tsr*(2-551)] ^c^ | this work |
| pRR22 | pJC3 [*tsr*(1-545) (L-1) *tsr*(1-551)] | this work |
| pRR48 | *bla* *lacI P_tac_ lacO_ideal_* [NdeI...BamHI...HindIII...KpnI] | [7] |
| pRR52 | pKG110 [*tsr*(1-545) (L-2) *tsr*(1-551)] [NdeI/AseI–NotI–KpnI] | this work |
| pRR53 | pRR48 [*tsr*(1-551)] [NdeI/AseI…KpnI] | [7] |
| pRR58 | pRR52 [*tsr*(1-545) (L-2) *tsr*(1-551/R69E)] | this work |
| pRR60 | pRR52 [*tsr*(1-545) (L-2) *tsr*(1-551/ R69E,T156K)] | this work |
| pRR64 | pRR52 [*tsr*(1-545/R69E) (L-2) *tsr*(1-551/R69E)] | this work |
| pRR65 | pRR52 [*tsr*(1-545/R69E) (L-2) *tsr*(1-551/T156K)] | this work |
| pRR67 | pRR52 [*tsr*(1-545/T156K) (L-2) *tsr*(1-551/R69E)] | this work |
| pRR68 | pRR52 [*tsr*(1-545/R69E) (L-2) *tsr*(1-551)] | this work |
| pRR71 | pRR52 [*tsr*(1-545/R69E,T156K) (L-2) *tsr*(1-551)] | this work |
| pRZ30 | *cat* *nahR P_nahG_* [*cheY-yfp* *cheZ-cfp*] | [19, 26] |
| pVS88 | pTrc99A *bla* *lacI* *P_trc_* [*cheY-yfp cheZ-cfp*] | [27] |

^a^ Plasmid-borne markers: *bla* = ampicillin resistance; *cat* = chloramphenicol resistance; *cfp* = cyan fluorescent protein; *lacI* = *lac* repressor (inducible by IPTG); *lacO* = *lac* operator; *lacO_ideal_* = perfectly palindromic *lac* operator that binds repressor more tightly than the native l*acO* for a more gradual induction ramp; *nahR* = salicylate-dependent activator of transcription from the *P_nahG_* promoter; *P_ara_* = arabinose-inducible promoter; *P_nahG_* = sodium salicylate-inducible promoter; *P_tac_* , *P_trc_* = hybrid *trp-lac* promoters; *yfp* = yellow fluorescent protein; *cheW*, *cheY*, *cheZ* = chemotaxis functions (see text). Expressed genes in plasmid constructs are listed in brackets; *tsr* constructs include the first and last codon/residue numbers in parentheses. Single-chain Tsr constructs include the designation of the linker polypeptide (L-1...L-6) that joins Tsr1 to Tsr2 (see Fig. 1). Missense mutations in a gene, *e.g.*, I377P, are listed after a slash and designated by the wild-type amino acid, the residue number, and the amino acid replacement in the mutant protein. Multiple mutations in a coding region are separated by commas. Restriction sites used in this work are listed in square brackets.

^b^ Derivative of pRR48 with EcoRV changed to EcoRI and KpnI changed to NheI.

^c^ The asterisk indicates an additional codon introduced in the cloning process at the beginning of the *tsr* gene. The extra amino acid at the N-terminus of Tsr is underlined: MLQK.
